# 330. Outpatient Parenteral Antimicrobial Therapy in People who Inject Drugs

**DOI:** 10.1093/ofid/ofad500.401

**Published:** 2023-11-27

**Authors:** Armani Hawes, Lisa Yanek, Megan E Buresh, Alia Bodnar, Sara C Keller

**Affiliations:** Johns Hopkins Hospital, Baltimore, Maryland; Johns Hopkins Hospital, Baltimore, Maryland; Johns Hopkins School of Medicine, Baltimore, Maryland; Johns Hopkins Hospital, Baltimore, Maryland; Johns Hopkins University School of Medicine, Baltimore, MD

## Abstract

**Background:**

People with opioid use disorder (OUD) make up a significant and increasing proportion patients requiring outpatient parenteral antimicrobial therapy (OPAT). Many of these patients do not receive OUD treatment during their index hospitalization, leading to missed opportunities for treatment, recurrent hospitalizations, worse outcomes, and increased costs. We describe predictors of addiction medicine consultation among people with OUD requiring OPAT, and the impact of addiction medicine consultation on receipt of medications for opioid use disorder (MOUD) after discharge.

**Methods:**

We reviewed records from 172 patients with OUD who were discharged on OPAT from Johns Hopkins Hospital and Johns Hopkins Bayview Medical Center 4/2020-9/2022. We described differences between those who received and did not receive inpatient addiction medicine consults by demographics, service (medicine vs surgical), type of insurance, type of infection, disposition, and addiction treatment.

**Results:**

The addiction medicine team was consulted for 120 patients and was not consulted for 46 patients. Addiction medicine was more likely to be consulted on younger patients (43.3 years vs 48.3 years, p=0.01), for patients with endovascular infections (23.3% vs 2.3%, p< 0.01) or bloodstream infections (31.9% vs 13.6%, p=0.02), and when the infectious organism was methicillin-resistant *Staphylococcus aureus* (40.7% vs 20.9%, p=0.02). When the addiction medicine team was consulted, patients were more likely to be discharged on MOUD, specifically buprenorphine (32.5% vs 17.4%, p< 0.01) or methadone (57.5% vs 39.1%, p< 0.01). Overall, most patients were discharged to a skilled nursing facility (SNF), but patients who received addiction medicine team consult were more likely to be discharged to SNF than home (92.9% vs 7.0%, p< 0.01). Patients discharged to SNF were less likely to have a 30 day readmission compared to patients discharged home (14.4% vs 32.0%, p=0.03).

**Figure:**
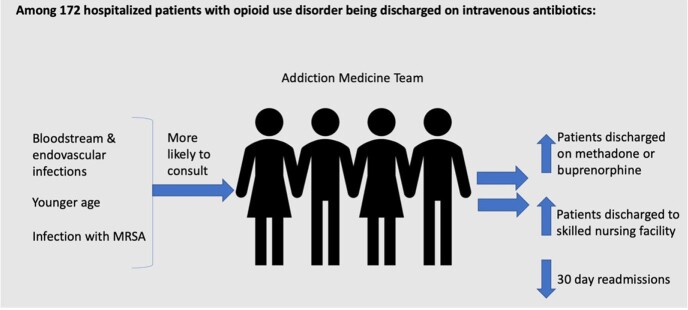

**Conclusion:**

Addiction medicine consultations were associated with an increased likelihood of evidence-based MOUD among patients with OUD requiring OPAT. By maximizing the resources of the addiction medicine team, we hope to improve outcomes for patients with opioid use disorder who require OPAT.

**Disclosures:**

**Sara C. Keller, MD, MPH, MSPH**, Pfizer: Advisor/Consultant

